# Effects of Simvastatin on Lipid Metabolism in Wild-Type Mice and Mice with Muscle PGC-1α Overexpression

**DOI:** 10.3390/ijms22094950

**Published:** 2021-05-07

**Authors:** Miljenko V. Panajatovic, Francois Singh, Stephan Krähenbühl, Jamal Bouitbir

**Affiliations:** 1 Division of Clinical Pharmacology & Toxicology, University Hospital of Basel, CH-4031 Basel, Switzerland; m.panajatovic@unibas.ch (M.V.P.); fzsingh@dundee.ac.uk (F.S.); stephan.kraehenbuehle@unibas.ch (S.K.); 2 Division of Pharmaceutical Technology, Department of Pharmaceutical Sciences, University of Basel, CH-4056 Basel, Switzerland; 3 Division of Molecular and Systems Toxicology, Department of Pharmaceutical Sciences, University of Basel, CH-4056 Basel, Switzerland

**Keywords:** simvastatin, PGC-1α, fatty acids, triglycerides, lipid droplets, perilipin 5, carnitine palmitoyltransferase 1b (CPT1b)

## Abstract

Previous studies suggest that statins may disturb skeletal muscle lipid metabolism potentially causing lipotoxicity with insulin resistance. We investigated this possibility in wild-type mice (WT) and mice with skeletal muscle PGC-1α overexpression (PGC-1α OE mice). In WT mice, simvastatin had only minor effects on skeletal muscle lipid metabolism but reduced glucose uptake, indicating impaired insulin sensitivity. Muscle PGC-1α overexpression caused lipid droplet accumulation in skeletal muscle with increased expression of the fatty acid transporter CD36, fatty acid binding protein 4, perilipin 5 and CPT1b but without significant impairment of muscle glucose uptake. Simvastatin further increased the lipid droplet accumulation in PGC-1α OE mice and stimulated muscle glucose uptake. In conclusion, the impaired muscle glucose uptake in WT mice treated with simvastatin cannot be explained by lipotoxicity. PGC-1α OE mice are protected from lipotoxicity of fatty acids and triglycerides by increased the expression of FABP4, formation of lipid droplets and increased expression of CPT1b.

## 1. Introduction

Despite the availability of new treatment options, statins currently remain the mainstay for the treatment against hypercholesterolemia [1]. They are used mainly in the prevention and the treatment of cardiovascular diseases associated with dyslipidemia [2,3,4,5]. Their mode of action is primarily via the inhibition of HMG-CoA reductase in the liver, the rate-limiting enzyme in the cholesterol biosynthesis pathway [6].

Statins have an excellent safety profile but are associated with skeletal muscle problems, a symptom complex called statin-associated muscle symptoms (SAMS) [7,8]. Affected patients have reported a spectrum of musculoskeletal complaints, ranging from muscle weakness and myalgia to rhabdomyolysis, potentially leading to death in rare cases [9]. SAMS are associated with all statins on the market, with a higher prevalence for lipophilic statins such as simvastatin, possibly because of their capacity to reach extrahepatic tissues such as skeletal muscle [10,11]. It is estimated that between 11% and 29% of the patients treated with statins show signs of muscle toxicity [7,8,9]. Skeletal muscle toxicity is therefore an important reason for medication nonadherence to statin treatment, which is a risk factor for cardiovascular outcomes [12]. Several mechanisms have been proposed causing statin-associated myopathy, including mitochondrial dysfunction [13,14,15], the activation of muscle atrophy and apoptosis [16,17] and impairment of the Akt/mTOR pathway [18].

In addition, insulin resistance and diabetes have been reported as adverse reactions of statins, which may increase the risk up to 30% in patients [19,20]. We have shown recently that the treatment of mice with simvastatin impairs the glucose uptake into skeletal muscle and induces insulin resistance [21,22]. Disturbances in muscle fatty acid (FA) metabolism have been reported to be associated with insulin resistance in patients with type 2 diabetes. Individuals with type 2 diabetes show an increased uptake of FA into skeletal muscle, which may be associated with insulin resistance [23]. Statins have been reported to cause lipid droplet accumulation in the skeletal muscle of certain patients, which suggests that statins can interfere with skeletal muscle FA and/or triglyceride metabolism [24]. In support of this assumption, lipophilic statins have been shown to inhibit FA metabolism in isolated rat skeletal muscle mitochondria [25]. These observations encouraged us to investigate the effect of simvastatin on skeletal muscle FA uptake and metabolism of mice in order to judge whether alterations in skeletal muscle metabolism could contribute to insulin resistance associated with simvastatin.

Peroxisome proliferator-activated receptor gamma (PPAR) coactivator 1 α (PGC-1α) is a transcription factor coactivator involved in the switch from glycolytic to oxidative metabolism. To achieve this metabolic switch, PGC-1α stimulates mitochondrial proliferation by activating the expression of mitochondrial genes and also stimulates FA transport and mitochondrial FA oxidation pathways [26]. Previous observations in human skeletal muscle myotubes and in mice indicated that the overexpression of PGC-1α stimulates FA uptake, triglyceride storage and mitochondrial FA oxidation [27,28,29]. We have shown recently that mice with muscle PGC-1α overexpression (OE mice) treated with simvastatin were protected from impaired skeletal muscle mitochondrial dysfunction and impaired exercise capacity, suggesting a role of PGC-1α in preventing simvastatin-associated myotoxicity [30]. On the other hand, we have previously demonstrated that statins impair mitochondrial β-oxidation in skeletal muscle cells [25].

Based on these considerations, we hypothesized that lipotoxicity may contribute to insulin resistance caused by simvastatin and that skeletal muscle PGC-1α overexpression may protect from simvastatin-induced lipotoxicity. To challenge this hypothesis, we investigated the effect of simvastatin on the plasma and skeletal lipid metabolism in wild-type mice and mice with muscle PGC-1α overexpression. 

## 2. Results

### 2.1. Characterization of the Animals

In order to estimate a possible genetic burden, we compared the body weight, food intake and water intake between WT and OE mice treated or not with simvastatin (Supplementary Figure S1A–C). There were no differences between WT and OE mice treated or not with simvastatin on body weight, food intake or water intake. As expected, the PGC-1α mRNA expression was higher in PGC-1α OE mice as compared to the respective WT mice (Supplementary Figure S2). Unexpectedly, simvastatin increased the PGC-1α mRNA expression in OE mice but not in WT mice. Different muscle weights were compared between mouse models and treatments to characterize the possible organ differences, as shown in Table 1. There were no significant differences between the mouse models, except for the quadriceps, where the mouse model factor was significant after the two-way ANOVA analysis. Moreover, simvastatin-treated OE mice had lower quadriceps muscle weights as compared to simvastatin-treated WT mice (Table 1). Simvastatin decreased the glucose uptake by the gastrocnemius muscle in WT mice but increased it in OE mice, as shown in Table 1. 

### 2.2. Cholesterol Concentrations in Plasma and Skeletal Muscle

**Figure 1:**
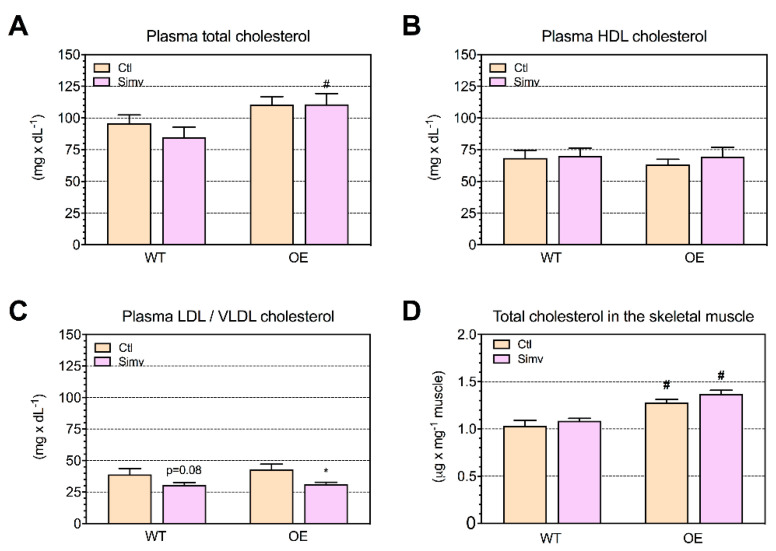
Cholesterol concentration in plasma and skeletal muscle. The cholesterol concentration was analyzed in mice after three weeks of treatment with water or simvastatin. Cholesterol was measured in plasma as total cholesterol (**A**), high-density lipoprotein (HDL) (**B**), low and very low-density lipoprotein (LDL and VLDL) (**C**) and cholesterol in the quadriceps muscle (**D**). Data are presented as the mean ± SEM of 6 animals per group. After the two-way ANOVA analysis, the animal model factor was significant (**A**,**D**), and the treatment factor was significant in (**C**). Symbols on the graphs are as follows: * *p* < 0.05 simvastatin-treated vs. their respective water-treated (control) mice and # *p* < 0.05 PGC-1α OE vs. WT mice of the same treatment group (water or simvastatin). Ctl, control; HDL, high-density lipoprotein; LDL, low-density lipoprotein; VLDL, very low-density lipoprotein; OE, PGC-1α overexpressing mice; Simv, simvastatin; WT, wild type.

Next, we investigated the effect of PGC-1α overexpression and simvastatin on plasma and skeletal muscle cholesterol. As shown in Figure 1A, PGC-1α overexpression caused an increase in total plasma cholesterol when compared to the respective water-treated groups, reaching statistical significance in simvastatin-treated mice. In contrast, neither PGC-1α overexpression nor simvastatin had an effect on the plasma HDL cholesterol concentration (Figure 1B). As expected, treatment with simvastatin decreased the plasma LDL/VLDL cholesterol concentration (Figure 1C). Similar to plasma, PGC-1α overexpression increased the skeletal muscle total cholesterol content, reaching statistical significance for both water-treated and simvastatin-treated mice vs. their respective WT mice (Figure 1D).

### 2.3. Triglyceride and Fatty Acid Concentrations in Plasma and Skeletal Muscle

**Figure 2:**
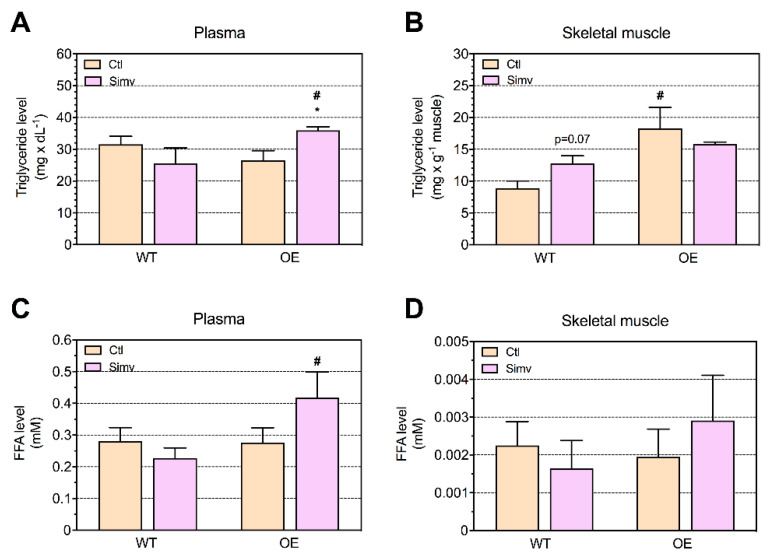
Triglyceride and fatty acid concentrations in the plasma and skeletal muscle. The triglyceride and free fatty acid (FFA) concentrations were analyzed in mice after three weeks of treatment with water (Ctl) or simvastatin. The triglyceride concentrations were measured in the plasma (**A**) and in the quadriceps muscle (**B**). The FFA concentrations were measured in the same plasma (**C**) and muscle (**D**) samples. Data are presented as the mean ± SEM of 6 animals per group. After the two-way ANOVA analysis, the animal model factor was significant in (**A**–**C**), the treatment factor in (**A**) and the interaction between treatment and animal model in (**A**). Symbols on the graphs are as follows: * *p* < 0.05 simvastatin-treated vs. their respective water-treated (control) mice and # *p* < 0.05 PGC-1α OE vs. WT mice of the same treatment group (water or simvastatin). Ctl, control; FFA, free fatty acid; OE, PGC-1α overexpressing mice; Simv, simvastatin; WT, wild type.

Subsequently, we investigated whether PGC-1α expression and/or simvastatin treatment affects the triglyceride and fatty acid concentration in plasma and/or skeletal muscle. As shown in Figure 2A, the plasma triglyceride concentration was not affected by PGC-1α expression. Simvastatin did not change the plasma triglyceride content in WT mice but increased it significantly in OE mice compared to water-treated or simvastatin-treated WT mice. In skeletal muscle, water-treated OE mice showed a doubling of the triglyceride content compared to the corresponding WT mice (Figure 2B). The treatment with simvastatin increased the skeletal muscle triglyceride content in WT mice by 40% without reaching significance (*p* = 0.07) and did not significantly affect the triglyceride content in OE mice.

As shown in Figure 2C, the plasma-free FA (FFA) concentration was not affected by the PGC-1α expression. The treatment with simvastatin did not significantly affect the FFA plasma concentration in WT mice. However, the plasma FFA was significantly higher in simvastatin-treated OE mice compared to simvastatin-treated WT mice (Figure 2C). Similarly to the triglyceride plasma concentration, PGC-1α overexpression did not affect the skeletal muscle FFA content (Figure 2D). Simvastatin had no significant effect on the FFA muscle content in both animal groups investigated.

### 2.4. Intermyofibrillar Lipid Droplets

**Figure 3:**
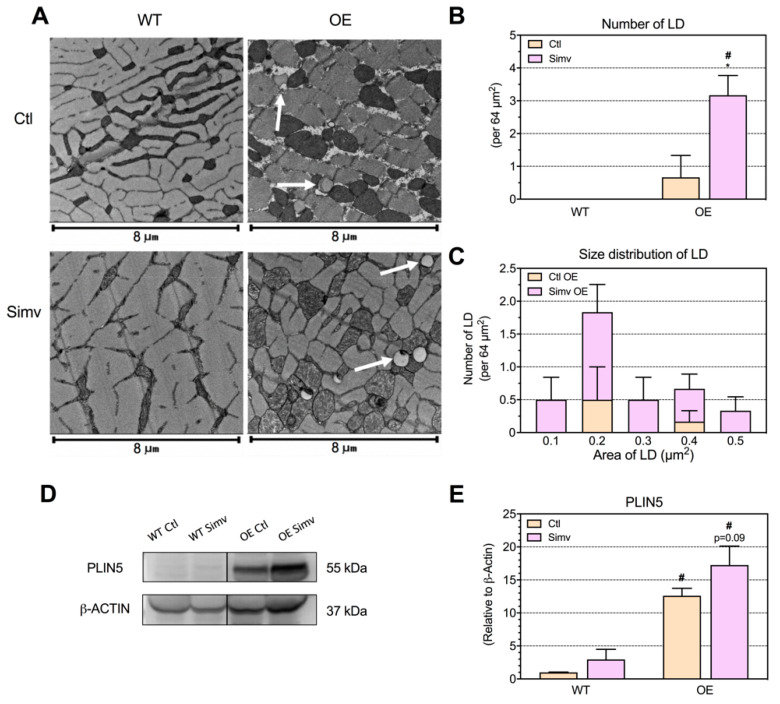
Lipid droplet content and PLIN5 protein expression in the skeletal muscle. The presence of lipid droplets (indicated by white arrows) in the quadriceps muscle was visualized with transmission electron microscopy (TEM) (**A**), counted in six animals per group (**B**) and the size determined in the same micrographs (**C**). Lipid accumulation was confirmed with perilipin 5 (PLIN5) protein expression (**D**) and the semi-quantitative analysis (**E**). Data are presented as the mean ± SEM of 6 micrographs for the TEM experiments and as mean ± SEM of 3 animals per group for Western blot. After the two-way ANOVA analysis, the animal model factor was significant for the number of lipid droplets (**B**) and PLIN5 protein expression (**E**). Symbols on the graphs are as follows: * *p* < 0.05 simvastatin-treated vs. their respective water-treated (control) mice and # *p* < 0.05 PGC-1α OE vs. WT mice of the same treatment group (water or simvastatin). Ctl, control; LD, lipid droplet; PLIN5, perilipin 5; OE, PGC-1α overexpressing mice; Simv, simvastatin; WT, wild type.

As a next step, we visualized lipid droplet accumulation within the muscle by transmission electron microscopy. The increased triglyceride skeletal muscle content in OE mice was associated with lipid droplet accumulation in the intermyofibrillar region, which was not visible in WT mice (Figure 3A). Lipid droplets were found in both OE water-treated and simvastatin-treated mice, with a higher number in simvastatin-treated mice (Figure 3B), whereas the size distribution was similar (0.24 ± 0.09 vs. 0.27 ± 0.13 µm^2^ in water-treated vs. simvastatin-treated OE mice, respectively; Figure 3C). To confirm the increase in lipid droplets in OE mice, we checked the protein expression of perilipin 5 (PLIN5) by immunoblotting, which is located at the surface of lipid droplets and can be used as an indirect measure of the lipid droplet content within the skeletal muscle [31]. As expected, OE mice showed a significant increase in PLIN5 protein expression as compared to WT mice (Figure 3D,E). OE mice treated with simvastatin showed a numerically higher mean value of PLIN5 when compared to water-treated OE mice.

### 2.5. Triglyceride Cleavage by Lipoprotein Lipase and Fatty Acid Uptake by CD36

**Figure 4:**
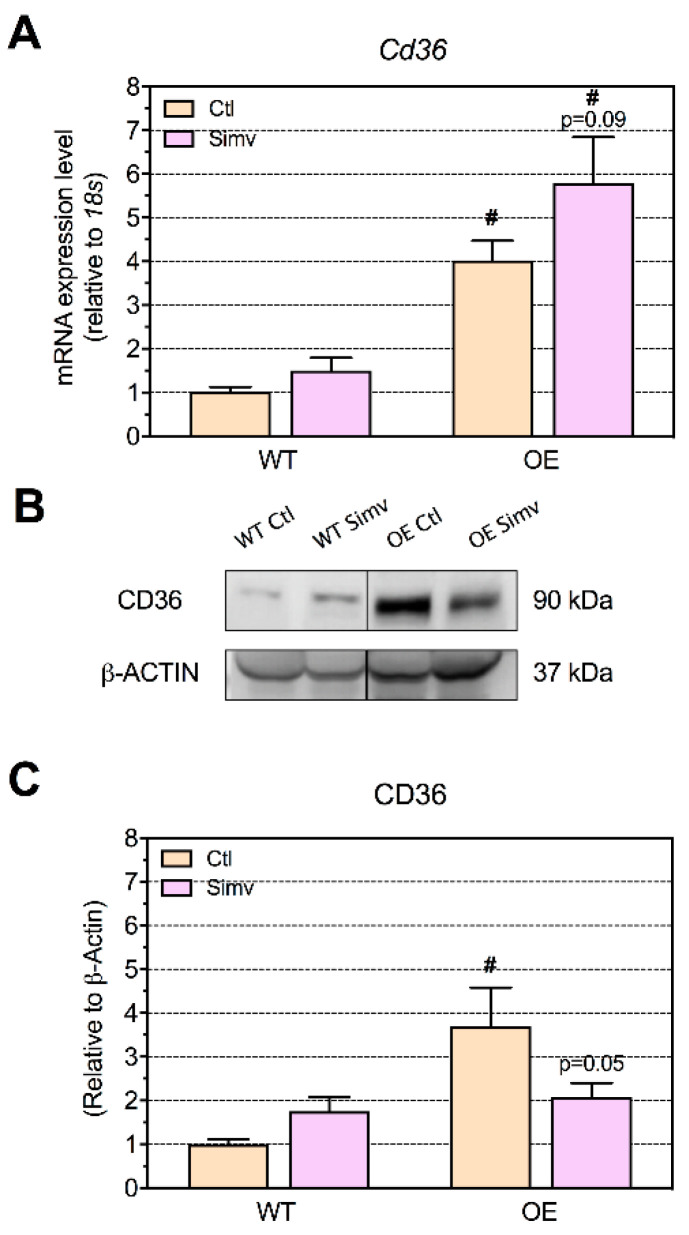
The expression of CD36. The mRNA expression of *Cd36* involved in fatty acid uptake in the quadriceps muscle (**A**). Representative Western blots are shown for the CD36 protein expression (**B**) and quantified in (**C**). Data are presented as the mean ± SEM of 8 animals per group for the mRNA expression and mean ± SEM of 3 animals for the Western blot. After the two-way ANOVA, the animal model factor was significant (**A**,**C**). Symbols on the graphs are as follows: # *p* < 0.05 PGC-1α OE vs. WT mice of the same treatment group (water or simvastatin). Ctl, control; OE, PGC-1α overexpressing mice; Simv, simvastatin; WT, wild type.

In order to explain the presence of lipid droplets, which were found in OE mice but not in WT mice, we first checked the expression of lipoprotein lipase and of the CD36 transporter involved in the fatty acid uptake [32]. We found an increased mRNA expression of lipoprotein lipase (*Lpl*) (Supplementary Figure S3) and of *Cd36* in the skeletal muscle of OE mice (both control and simvastatin-treated groups) as compared to WT (Figure 4A). The protein expression of CD36 was increased in water-treated OE mice as compared to WT mice, thus confirming the mRNA expression results (Figure 4B,C). Simvastatin increased the mRNA and protein expression of CD36 in WT numerically (Figure 4A,C), suggesting an increased fatty acid uptake by simvastatin in WT mice. In OE mice, simvastatin decreased the protein expression of CD36 by trend (*p* = 0.05) (Figure 4B,C).

### 2.6. FA Uptake and Activation by FATP4

**Figure 5:**
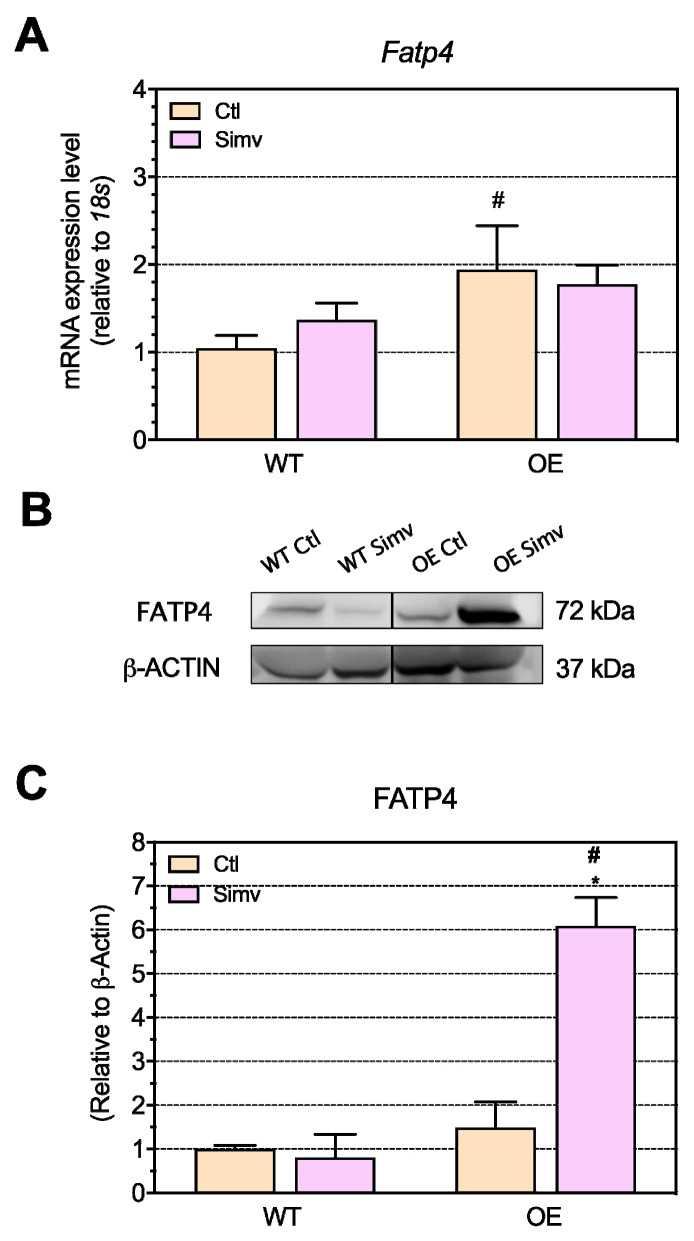
The mRNA and protein expression of fatty acid uptake (FATP4). The *Fatp4* mRNA expression in the quadriceps muscle involved in fatty acid transport and activation (**A**). Representative Western blots are shown in (**B**) and quantified in (**C**). Data are presented as the mean ± SEM of 8 animals per group for the mRNA expression and mean ± SEM of 3 animals for the Western blot. After the two-way ANOVA analysis, the treatment factor and the interaction between the treatment and animal model were significant in (**C**). Moreover, the animal model factor was significant in (**A**,**C**). Symbols on the graphs are as follows: * *p* < 0.05 simvastatin-treated vs. respective water-treated (control) mice and # *p* < 0.05 PGC-1α OE vs. WT mice of the same treatment group (water or simvastatin). Ctl, control; FATP4, fatty acid transport protein 4; OE, PGC-1α overexpressing mice; Simv, simvastatin; WT, wild type.

A second protein involved in FA uptake and also activation is fatty acid transport protein 4 (FATP4) [33]. The mRNA expression of *Fatp4* was significantly increased in water-treated OE mice as compared to water-treated WT mice (Figure 5A). The treatment with simvastatin revealed no significant effect on the mRNA expression of *Fatp4*. In water-treated mice, there was no significant difference of the FATP4 protein expression between the mouse models investigated (Figure 5B,C). However, simvastatin increased the FATP4 protein expression six-fold in OE mice.

### 2.7. Intracellular Transport of Fatty Acids through Fatty Acid Binding Protein 4 (FABP4)

**Figure 6:**
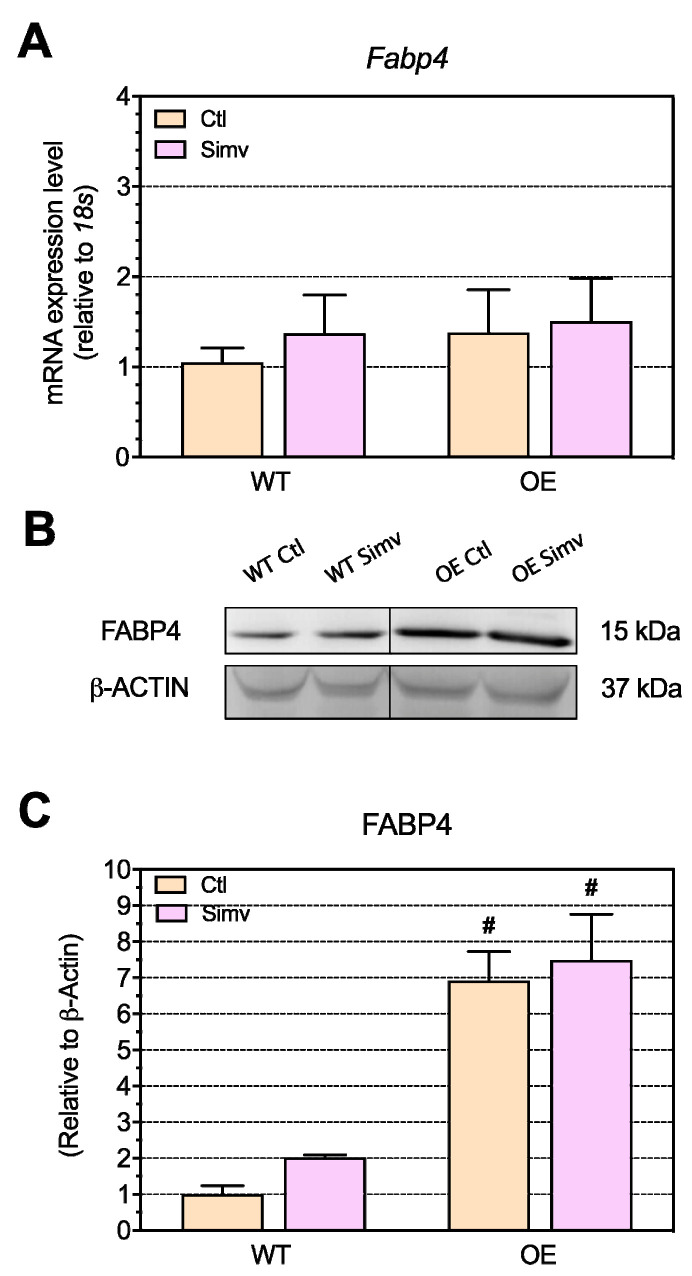
The mRNA and protein expression of the fatty acid transport (FABP4). The fatty acid-binding protein 4 (Fapb4) mRNA (**A**) and protein expression (FABP4) (**B**,**C**) was checked for the fatty acid transport and trafficking capabilities in the quadriceps muscle. Data are presented as the mean ± SEM of 8 animals per group for the mRNA expression and mean ± SEM of 3 animals for the Western blot. After the two-way ANOVA analysis, the animal model factor was significant in (**C**). Symbols on the graphs are as follows: * *p* < 0.05 simvastatin-treated vs. respective water-treated (control) mice and # *p* < 0.05 PGC-1α OE vs. WT mice of the same treatment group (water or simvastatin). Ctl, control; FABP4, fatty acid binding protein 4; OE, PGC-1α overexpressing mice; Simv, simvastatin; WT, wild type.

Due to increased uptake of fatty acids in OE mice, as suggested by the increased expression of CD36 and FATP4, we checked the expression of fatty acid-binding protein 4 (FABP4), which is involved in FA intracellular trafficking [34]. Regarding the mRNA expression of *Fabp4*, there were no significant differences between the mouse models used (Figure 6A). Furthermore, simvastatin had no significant effect on the *Fabp4* mRNA expression. Compared to the respective WT mice, water-treated and simvastatin-treated OE mice showed a significant increase in the FABP4 protein expression (Figure 6B,C). Simvastatin doubled the FABP4 protein expression in WT mice, but this increase did not reach statistical significance.

### 2.8. Fatty Acid Transport into Mitochondria

**Figure 7:**
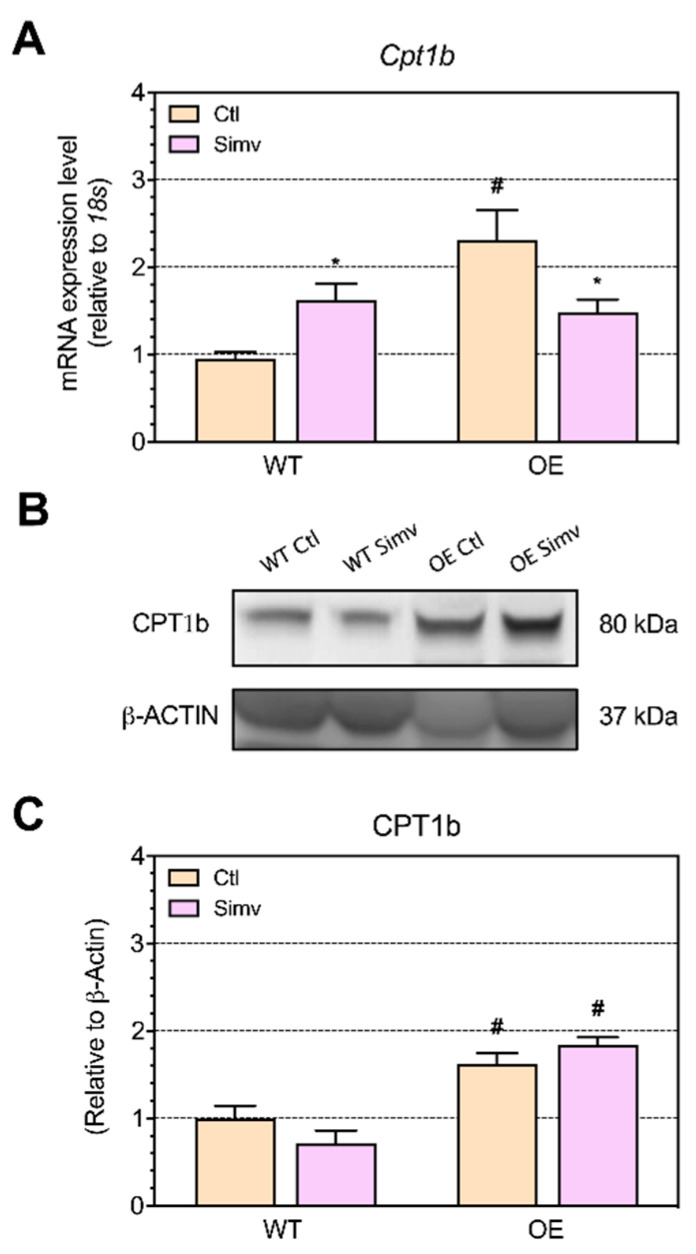
The mRNA and protein expression of CPT1b. We assessed the mRNA and protein expression of CPT1b in gastrocnemius muscle. The CPT1b mRNA expression is shown in (**A**) and the protein expression in (**C**,**B**). Data are presented as the mean ± SEM of 8 animals per group for the mRNA expression and 3 animals per group for the protein expression. After the two-way ANOVA analysis, the treatment factor was significant (**A**), the animal model factor significant for (**A**,**C**) and the interaction between the treatment and animal model significant in (**B**). Symbols on the graphs are as follows: * *p* < 0.05 simvastatin-treated vs. respective water-treated (control) mice and # *p* < 0.05 PGC-1α OE vs. WT mice of the same treatment group (water or simvastatin). Ctl, control; OE, PGC-1α overexpressing mice; Simv, simvastatin; WT, wild type.

Fatty acid transport into the mitochondria is realized through the carnitine shuttle, whereby carnitine palmitoyltransferase 1 (CPT1) is considered to be rate-limiting [35]. We determined the mRNA and protein expression of CPT1b, which is located in the outer mitochondrial membrane and produces the corresponding carnitine derivative from long-chain acyl-CoAs [35]. The mRNA expression of *Cpt1b* was increased in water-treated OE mice as compared to WT mice (Figure 7A). Simvastatin increased the expression of *Cpt1b* in WT mice but decreased the *Cpt1b* mRNA expression in OE mice. PGC-1α overexpression increased the protein expression of CPT1b (Figure 7B,C). Simvastatin did not significantly affect the CPT1b protein expression.

## 3. Discussion

The aim of the current study was to investigate the effect of simvastatin and skeletal muscle PGC-1α overexpression on the plasma and muscle lipid metabolism and its relation to skeletal muscle glucose uptake as a marker of insulin sensitivity.

When comparing the two mouse models investigated, water-treated PGC-1α OE mice showed a higher cholesterol and triglyceride content, accumulation of lipid droplets and increased perilipin 5 and CPT1b expression in skeletal muscle compared to water-treated WT mice. These alterations are in line with the previous publication from Choi et al., where the authors described an increase in the skeletal muscle triglyceride content in PGC-1α overexpression compared to the control mice [36]. However, in the study of Choi et al., the plasma FFA concentration was higher in PGC-1α OE than WT mice, which was not the case in the current investigation. Since the mice were fasted in the study of Choi et al. but had free access to food in the current investigation, this discrepancy between the two studies can be explained by the different metabolic state of the animals. Since the skeletal muscle triglyceride content was higher in OE mice compared to WT mice, we visualized the presence of lipid droplets in skeletal muscle of the mice by electron microscopy. Indeed, we could confirm the presence of lipid droplets in OE mice but not in WT mice. The increase in the skeletal muscle triglyceride content in mice with muscle PGC-1α overexpression has been explained in previous investigations by a rise in the expression of CD36, fatty acid synthase (FAS) and enzymes responsible for triglyceride formation [28,36]. These findings suggested that the induction of fatty acid uptake, fatty acid synthesis and triglyceride formation are responsible for lipid accumulation in skeletal muscle of PGC-1α OE mice. In the current study, we could confirm an increase in the mRNA and protein expression of CD36 in OE mice, whereas the protein expression of FATP4, an additional fatty acid transport protein that we assessed [33], was not significantly affected by the PGC-1α expression. However, regarding the strong effect of PGC-1α overexpression on the expression of *Lpl* and CD36, we also regard an increased cellular uptake of FA as a major driving force for skeletal muscle lipid accumulation in OE mice.

Simvastatin affected significantly the lipid metabolism in OE mice. As expected, it decreased the plasma LDL/VLDL concentration, and it increased the cholesterol content in skeletal muscle. Regarding the fatty acid/triglyceride metabolism, simvastatin increased the accumulation of lipid droplets in the skeletal muscle, increased the protein expression of FATP4 and decreased the mRNA expression of *Cpt1b*. Furthermore, it increased the plasma fatty acid concentration by trend. The increase in the skeletal muscle lipid droplet content by simvastatin in OE mice could therefore be explained best by the increased transport and activation of FA into the skeletal muscle and by reduced transport into the mitochondrial matrix with the subsequent impairment of FA degradation.

Glucose uptake by the skeletal muscle in water-treated OE mice was numerically decreased but statistically not different from water-treated WT mice. This suggests that, despite the accumulation of lipid droplets, the skeletal muscle insulin sensitivity was not significantly impaired in OE mice. The formation of lipid droplets can be regarded as a measure to avoid the toxicity of lipids accumulating in cells [37]. Lipid droplets are dynamic structures with a triglyceride and cholesterol core surrounded by phospholipids and a protein layer, which is important for the metabolism of the lipids contained within the droplets [38]. The proteins contained in this layer are, for instance, enzymes involved in triglyceride synthesis, proteins of the perilipin family, including PLIN5, and lipases such as the adipose tissue triacylglycerol lipase. PLIN5 has a high expression in oxidative tissues, such as heart and skeletal muscle, and promotes the formation of lipid droplets [31]. The skeletal muscle overexpression of PGC-1α has already been shown to increase the expression of PLIN5 [39]. The phospholipid and the protein layer protect cell organelles from potentially toxic triglycerides and cholesterol esters. A high expression of PLIN5, which promotes lipid droplet formation, can therefore be regarded as a protective measure of the cells to avoid lipotoxicity [37,40]. In addition, PGC-1α overexpression was also associated with a high expression of FABP4 in the skeletal muscle of OE mice. After entering the cells, fatty acids are bound to fatty acid-binding proteins, which allow transport within the cell and also diminishes their toxicity [34]. The increase in the expression of FABP4 in myocytes of OE mice can therefore be regarded as an additional measure of myocytes to avoid lipotoxicity. The increase in CPT1b expression in the skeletal muscle of PGC-1α OE mice can be interpreted as a consequence of PGC-1α overexpression, which stimulates mitochondrial biogenesis [26]. Since CPT1b controls the import and breakdown of fatty acids [35], a high activity of CPT1b also helps to control the intracellular fatty acid concentration.

Lipotoxicity is a well-known reason for decreased insulin sensitivity, which impairs the skeletal muscle glucose uptake [37,40]. The treatment with simvastatin increased the muscle lipid droplet content of OE mice but, at the same time, also increased the glucose uptake into the skeletal muscle, indicating maintained or even improved insulin sensitivity, despite the lipid accumulation. This apparent paradoxical finding underscores the importance of lipid droplet formation in the case of the increased availability of fatty acids in order to protect cells from their potentially toxic effects.

In comparison to OE mice, the effect of simvastatin on the lipid metabolism was minimal in WT mice. Simvastatin significantly increased the *Cpt1b* mRNA expression but without affecting the CPT1b protein expression and had otherwise no significant effects on the parameters determined. Simvastatin increased by trend the triglyceride content and the expression of PLIN5, CD36 and FABP4. These changes are similar to those observed in OE mice but did not reach significance. The mechanism of these changes is not clear but is clearly not related to PGC-1α, since simvastatin did not alter the PGC-1α muscle expression. However, in contrast to OE mice, simvastatin decreased significantly the glucose transport into the skeletal muscle of the WT mice. Since simvastatin did not cause lipid accumulation in the skeletal muscle of the WT mice, lipotoxicity appears not to be involved in the impairment of insulin sensitivity by simvastatin in WT mice. We showed recently that simvastatin impairs the activation of Akt, which has an important role in the post-receptor signaling of insulin, and IGF-1, explaining the simvastatin-associated impairment of insulin sensitivity [22,41]. PGC-1α overexpression protects mice from simvastatin-associated insulin resistance [21].

In conclusion, in WT mice, simvastatin has only minor effects on the muscle lipid metabolism but decreases the glucose uptake. Reduced glucose uptake by simvastatin in WT mice cannot be explained by lipotoxicity and may be related to alterations in insulin signaling. PGC-1α overexpression leads to skeletal muscle lipid droplet accumulation without the impairment of skeletal muscle glucose uptake. Simvastatin increases the lipid droplet accumulation in PGC-1α OE mice and improves the skeletal muscle glucose uptake. Protection from lipotoxicity in PGC-1α OE mice is mainly due to the increased expression of PLIN5 and FABP4, which traps the potentially toxic FA and triglycerides.

## 4. Materials and Methods

### 4.1. Ethical Approval of the Animal Study

In Vivo experiments were performed in accordance with the National Institute of Health guide for the care and use of Laboratory animals (NIH Publication No. 8023, revised 1978). Experiments were reviewed and accepted by the cantonal veterinary authority of Basel (License number 2847, approved on 21 July 2016). For the purpose of this study, male wild-type mice (WT) and mice overexpressed PGC-1α in the muscle (OE) aged between 15–18 weeks. Mice were kept under 22 ± 2 °C on a 12-h dark or light cycle with access to food and water ad libitum. Mice received a standard pellet chow (Kliba Futter 3436, Kaiseraugst, Switzerland). The diet was composed of 35% carbohydrates, 18.5% proteins and 4.5% fat. Mice breeding pairs for the generation of OE male mice were kindly provided by Prof. Handschin (Basel University, Basel, Switzerland). OE mice that overexpressed PGC-1α were developed using the DNA microinjection technique by Ppargc1a gene insertion under the myogenin promotor [42]. Male mice from the OE mouse line, without genetic mutations, were used as WT mice.

### 4.2. Simvastatin Administration

Mice were randomly divided into following four groups: (1) WT control animals treated with water (Ctl; *n* = 10), (2) WT animals treated with simvastatin 5 mg × kg^−1^ × day^−1^ (Simv; *n* = 10), (3) OE mice treated with water (*n* = 10) and (4) OE mice treated with simvastatin 5 mg × kg^−1^ × day^−1^ (*n* = 10). Mice were treated every morning (between 8 and 10 a.m.) by oral gavage for three weeks. The dose of 5 mg × kg^−1^ used for the mice achieved similar peak plasma concentrations in comparison to human patients treated with 40 mg of simvastatin [30]. Welfare of the mice was inspected daily before the treatment by measuring body weight, food and water intake.

### 4.3. Sample Collection

After 3 weeks of treatment with simvastatin, mice were anesthetized with an intraperitoneal injection of ketamine (160 mg/kg, Ketasol, Graeub, Bern, Switzerland) and xylazine (20 mg/kg, Rompun, Bayer, Leverkusen, Germany). Blood was removed from the heart and placed in a tube coated with EDTA. We collected plasma samples after centrifugation at 3000 rpm for 15 min at 4 °C. Skeletal muscle (gastrocnemius and quadriceps) were immediately collected and snap-frozen in liquid nitrogen. Plasma and skeletal muscle samples were stored at −80 °C for later analysis.

### 4.4. In Vivo Glucose Transport into Skeletal Muscle

The transport of glucose into skeletal muscle was determined in water-treated and simvastatin-treated WT and PGC-1α OE mice fasted overnight to ensure low-plasma insulin concentrations, as described previously [21]. Briefly, mice received an i.p. injection of glucose and ^3^H-deoxyglucose, followed by the determination of the area under the curve (AUC) of ^3^H-deoxyglucose in plasma over 30 min and of the accumulation of phosphorylated ^3^H-deoxyglucose in the gastrocnemius muscle 30 min after i.p. injection. These parameters allowed the determination of skeletal muscle deoxyglucose clearance and of skeletal muscle glucose uptake by multiplication with the plasma glucose concentration.

### 4.5. Quantification of Cholesterol, Triglyceride and Free Fatty Acid Contents

Cholesterol, triglyceride and free fatty acid contents were analyzed in the plasma and quadriceps muscle isolated from mice after three weeks of treatment. Muscle samples were homogenized with a microdismembrator for 30 s at 3000 rpm (Sartorius Stedim Biotech, Aubagne, France). For the cholesterol and triglyceride contents, muscles were lysed in lysis buffer from the assay kit (ab65390 and ab178780, Abcam, Cambridge, UK) and measured according to the manufacturer’s instructions. For the free fatty acid content, muscles were lyzed in chloroform containing 1% Triton X, centrifuged at 4 °C for 5 min at 16,000× *g* and the supernatant was vacuum-dried at 50 °C for 30 min. Dried samples were reconstituted and measured according to the manufacturer’s instructions (ab65341, Abcam, Cambridge, UK). Cholesterol, triglyceride and free fatty acid contents were measured directly from the plasma according to the manufacturer’s instructions.

### 4.6. Electron Transmission Microscopy

Immediately during sacrifice, the quadriceps muscle was freshly dissected and fixed in fixative solution containing 2.5% glutaraldehyde and 2% paraformaldehyde in a PIPES buffer (0.1 M at pH 7) overnight at 4 °C. Post-fixation was done in 1% buffered osmium tetroxide for 1 h at 4 °C. Samples were dehydrated in solvents with increasing ethanol concentrations. Embedding was done in Epon 812 resin and hardened in an oven at 60 °C for 48 h for further sectioning. Ultrathin sections were impregnated with uranyl acetate and lead citrate. Sections were analyzed using a FEI Tecnai T12 transmission electron microscope and recorded using a TVIPS F416 CMOS digital camera. Micrographs were randomly taken from transversal intermyofibrillar sections at the nonoverlapping 64-μm^2^ regions. Fiji software (https://imagej.net/Fiji/Downloads (accessed on 10 March 2021), version: 2.1.0/1.53c) was used for the quantitative analysis of lipid droplets as described [43].

### 4.7. Western Blots

Quadriceps muscle samples (50 mg) were first homogenized with a microdismembrator for 30 s at 3000 rpm (Sartorius Stedim Biotech, Aubagne, France) and lysed on ice in PhosphoSafe™ Extraction Reagent. Homogenates were then centrifuged at 16,000× *g* at 4 °C for 10 min, and the protein was determined in the supernatant with a Pierce BCA protein assay kit (Thermo Fisher Scientific, Waltham, MA, USA). The amount of 80 μg of protein was loaded into the wells of the NuPAGE 4–12% Bis-Tris gel (Life technologies, Rockville, MD, USA). Gel was run at 140 V and electroblotted to the nitrocellulose membrane using the eBlot™ L1 Wet Transfer System (GenScript, Piscataway, NJ, USA). Proteins were immunodetected using antibodies against PLIN5 (1:1000, 26951-1-AP, Proteintech, Rosemont, IL, USA), CD36 (1:1000, ab133625, Abcam, Cambridge, UK), FATP4 (1:1000, ab200353, Abcam, Cambridge, UK), FABP4 (1:1000, ab92501, Cambridge, UK, Abcam), CPT1b (1:1000, 22170-1-AP, Proteintech, Rosemont, IL, USA) and β-actin (1:20,000, sc-47778, Santa Cruz Biotechnology, Dallas, TX, USA). Membranes were then probed with secondary HRP-conjugated antibodies against rabbit (1:2000, sc-2004 Santa Cruz Biotechnology, Dallas, TX, USA ) or mouse (1:2000, sc-516102, Santa Cruz Biotechnology, Dallas, TX, USA); after which, a chemiluminescent substrate (Clarity Western ECL substrate; Bio-Rad Laboratories, Hercules, CA, USA) was added to visualize the bands. Protein expression was quantified using the Fusion Pulse TS device (Vilber Lourmat, Oberschwaben, Germany).

### 4.8. Quantitative Real-Time PCR

Total RNA was isolated from gastrocnemius (40 mg) using the RNeasy Fibrous Tissue Mini Kit (QIAGEN Gmbh, Hilden, Germany), according to the manufacturer’s instructions. For the reverse transcription reaction, cDNA was synthetized from 0.5 µg of total RNA with the Omniscript RT kit (QIAGEN GmbH, Hilden, Germany). Next, cDNA was mixed together with forward and reverse 0.3-µM primers and SYBR Green (Roche Diagnostics, Mannheim, Germany) as a fluorescent dye for the measurements of the duplex DNA formation. The real-time PCR measurements were measured in triplicate with the ViiA™ 7 Real-Time PCR System (Applied Biosystems, Waltham, MA, USA). The sequences of the primer sets used are listed in Table 2. Quantification of the gene expression was performed as described previously [44], using the *18s* gene as the internal control.

### 4.9. Statistical Analysis

Data are represented as the mean ± SEM. Statistical analyses were performed by 2-way ANOVA, followed by Fisher’s LSD post-test without correction for the comparison of multiple means using GraphPad Prism 8 (Graph Pad Software, San Diego, CA, USA) [45]. Significance was set at *p* < 0.05.

## Figures and Tables

**Table 1:** Muscle weights and glucose uptake of WT and OE treated or not with simvastatin.

Mouse Model Treatment	WT Ctl	WT Simv	OE Ctl	OE Simv
Gastrocnemius	5.10 ± 0.15	5.09 ± 0.23	5.20 ± 0.09	5.40 ± 0.12
Soleus	0.26 ± 0.02	0.29 ± 0.01	0.3 ± 0.02	0.27 ± 0.03
Quadriceps	7.09 ± 0.35	7.39 ± 0.26	6.45 ± 0.31	6.55 ± 0.27#
EDL	0.30 ± 0.03	0.33 ± 0.03	0.30 ± 0.03	0.33 ± 0.01
Glucose uptake	2.3 ± 0.5	1.2 ± 0.3 *	1.4 ± 0.2	2.8 ±0. 5 *#

Values for the muscle weight are shown in (weight per body weight in mg × g^−1^) and for glucose uptake (µmol × min^−1^ × 100 g^−1^ wet weight). All values are expressed as mean ± SEM with *n* = 4–10 per group. Abbreviations: EDL, extensor digitorum longus; OE, muscle PGC-1α overexpression mice; WT, wild-type mice. * *p* < 0.05 simvastatin-treated vs. respective water-treated (control) mice and # *p* < 0.05 between the control or simvastatin groups of OE mice and WT mice.

**Table 2:** Primer list for the quantitative real-time PCR amplification.

Target Gene	Forward Primer 5′ → 3′ Reverse Primer 5′ → 3′
*Cd36*	GGCAAAGAACAGCAGCAAAAT TGGCTAGATAACGAACTCTGTATGTGT
*Fatp4*	GTGAGATGGCCTCAGCTATC GAAGAGGGTCCAGATGCTCT
*Fabp4*	GAACCTGGAAGCTTGTCTTCG ACCAGCTTGTCACCATCTCG
*Lpl*	GTGGCCGCAGCAGACGCAGGAAGA CATCCAGTTGATGAATCTGGCCAC
*Cpt1b*	ATCATGTATCGCCGCAAACT CCATCTGGTAGGAGCACATGG
*Ppargc1a*	AATGCAGCGGTCTTAGCACT ACGTCTTTGTGGCTTTTGCT
*18s*	AGTCCCTGCCCTTTGTACACA CGATCCGAGGGCCTCACTA

## Data Availability

The data presented in this study are available on request from the corresponding author.

## Supplementary Materials

The following are available online at https://www.mdpi.com/article/10.3390/ijms22094950/s1: Figure S1: Physiological characteristics over the course of the treatment, Figure S2: Expression of *PGC-1α* mRNA in skeletal muscle, and Figure S3: mRNA expression of lipoprotein (*Lpl*) in skeletal muscle.

## References

[1] Grundy S.M. (1988). HMG-CoA reductase inhibitors for treatment of hypercholesterolemia. N. Engl. J. Med..

[2] Cholesterol Treatment Trialists’ (CTT) Collaboration (2019). Efficacy and safety of statin therapy in older people: A meta-analysis of individual participant data from 28 randomised controlled trials. Lancet.

[3] Baigent C., Blackwell L., Emberson J., Holland L.E., Reith C., Bhala N., Peto R., Barnes E.H., Keech A., Cholesterol Treatment Trialists’ (CTT) Collaboration (2010). Efficacy and safety of more intensive lowering of LDL cholesterol: A meta-analysis of data from 170,000 participants in 26 randomised trials. Lancet.

[4] Fulcher J., O’Connell R., Voysey M., Emberson J., Blackwell L., Mihaylova B., Simes J., Collins R., Kirby A., Cholesterol Treatment Trialists’ Collaboration (2015). Efficacy and safety of LDL-lowering therapy among men and women: Meta-analysis of individual data from 174,000 participants in 27 randomised trials. Lancet.

[5] Mihaylova B., Emberson J., Blackwell L., Keech A., Simes J., Barnes E.H., Voysey M., Gray A., Collins R., Cholesterol Treatment Trialists’ Collaboration (2012). The effects of lowering LDL cholesterol with statin therapy in people at low risk of vascular disease: Meta-analysis of individual data from 27 randomised trials. Lancet.

[6] Istvan E.S., Deisenhofer J. (2001). Structural mechanism for statin inhibition of HMG-CoA reductase. Science.

[7] Bouitbir J., Sanvee G.M., Panajatovic M.V., Singh F., Krahenbuhl S. (2020). Mechanisms of statin-associated skeletal muscle-associated symptoms. Pharmacol. Res..

[8] Ward N.C., Watts G.F., Eckel R.H. (2019). Response by Ward et al to Letter Regarding Article, “Statin Toxicity: Mechanistic Insights and Clinical Implications”. Circ. Res..

[9] Sakamoto K., Kimura J. (2013). Mechanism of statin-induced rhabdomyolysis. J. Pharmacol Sci.

[10] Banach M., Rizzo M., Toth P.P., Farnier M., Davidson M.H., Al-Rasadi K., Aronow W.S., Athyros V., Djuric D.M., Ezhov M.V. (2015). Statin intolerance—An attempt at a unified definition. Position paper from an International Lipid Expert Panel. Arch. Med. Sci..

[11] Bitzur R., Cohen H., Kamari Y., Harats D. (2013). Intolerance to statins: Mechanisms and management. Diabetes Care.

[12] Chowdhury R., Khan H., Heydon E., Shroufi A., Fahimi S., Moore C., Stricker B., Mendis S., Hofman A., Mant J. (2013). Adherence to cardiovascular therapy: A meta-analysis of prevalence and clinical consequences. Eur. Heart J..

[13] Bouitbir J., Charles A.L., Echaniz-Laguna A., Kindo M., Daussin F., Auwerx J., Piquard F., Geny B., Zoll J. (2012). Opposite effects of statins on mitochondria of cardiac and skeletal muscles: A ‘mitohormesis’ mechanism involving reactive oxygen species and PGC-1. Eur. Heart J..

[14] Schirris T.J., Renkema G.H., Ritschel T., Voermans N.C., Bilos A., van Engelen B.G., Brandt U., Koopman W.J., Beyrath J.D., Rodenburg R.J. (2015). Statin-Induced Myopathy Is Associated with Mitochondrial Complex III Inhibition. Cell Metab..

[15] Singh F., Zoll J., Duthaler U., Charles A.L., Panajatovic M.V., Laverny G., McWilliams T.G., Metzger D., Geny B., Krahenbuhl S. (2019). PGC-1beta modulates statin-associated myotoxicity in mice. Arch. Toxicol..

[16] Bouitbir J., Singh F., Charles A.L., Schlagowski A.I., Bonifacio A., Echaniz-Laguna A., Geny B., Krahenbuhl S., Zoll J. (2016). Statins Trigger Mitochondrial Reactive Oxygen Species-Induced Apoptosis in Glycolytic Skeletal Muscle. Antioxid. Redox Signal..

[17] Cao P., Hanai J., Tanksale P., Imamura S., Sukhatme V.P., Lecker S.H. (2009). Statin-induced muscle damage and atrogin-1 induction is the result of a geranylgeranylation defect. FASEB J..

[18] Jaskiewicz A., Pajak B., Litwiniuk A., Urbanska K., Orzechowski A. (2018). Geranylgeraniol Prevents Statin-Dependent Myotoxicity in C2C12 Muscle Cells through RAP1 GTPase Prenylation and Cytoprotective Autophagy. Oxid. Med. Cell Longev..

[19] Crandall J.P., Mather K., Rajpathak S.N., Goldberg R.B., Watson K., Foo S., Ratner R., Barrett-Connor E., Temprosa M. (2017). Statin use and risk of developing diabetes: Results from the Diabetes Prevention Program. BMJ Open Diabetes Res. Care.

[20] Ridker P.M., Pradhan A., MacFadyen J.G., Libby P., Glynn R.J. (2012). Cardiovascular benefits and diabetes risks of statin therapy in primary prevention: An analysis from the JUPITER trial. Lancet.

[21] Panajatovic M.V., Singh F., Krahenbuhl S., Bouitbir J. (2020). Simvastatin Impairs Glucose Homeostasis in Mice Depending on PGC-1alpha Skeletal Muscle Expression. Biomedicines.

[22] Sanvee G.M., Panajatovic M.V., Bouitbir J., Krahenbuhl S. (2019). Mechanisms of insulin resistance by simvastatin in C2C12 myotubes and in mouse skeletal muscle. Biochem. Pharmacol..

[23] Lopaschuk G.D. (2016). Fatty Acid Oxidation and Its Relation with Insulin Resistance and Associated Disorders. Ann. Nutr. Metab..

[24] Phillips P.S., Haas R.H., Bannykh S., Hathaway S., Gray N.L., Kimura B.J., Vladutiu G.D., England J.D., Scripps Mercy Clinical Research Center (2002). Statin-associated myopathy with normal creatine kinase levels. Ann. Intern. Med..

[25] Kaufmann P., Torok M., Zahno A., Waldhauser K.M., Brecht K., Krahenbuhl S. (2006). Toxicity of statins on rat skeletal muscle mitochondria. Cell Mol. Life Sci..

[26] Cheng C.F., Ku H.C., Lin H. (2018). PGC-1alpha as a Pivotal Factor in Lipid and Metabolic Regulation. Int. J. Mol. Sci..

[27] Huang T.Y., Zheng D., Houmard J.A., Brault J.J., Hickner R.C., Cortright R.N. (2017). Overexpression of PGC-1alpha increases peroxisomal activity and mitochondrial fatty acid oxidation in human primary myotubes. Am. J. Physiol. Endocrinol. Metab..

[28] Summermatter S., Baum O., Santos G., Hoppeler H., Handschin C. (2010). Peroxisome proliferator-activated receptor {gamma} coactivator 1{alpha} (PGC-1{alpha}) promotes skeletal muscle lipid refueling in vivo by activating de novo lipogenesis and the pentose phosphate pathway. J. Biol. Chem..

[29] Summermatter S., Shui G., Maag D., Santos G., Wenk M.R., Handschin C. (2013). PGC-1alpha improves glucose homeostasis in skeletal muscle in an activity-dependent manner. Diabetes.

[30] Panajatovic M.V., Singh F., Roos N.J., Duthaler U., Handschin C., Krahenbuhl S., Bouitbir J. (2020). PGC-1alpha plays a pivotal role in simvastatin-induced exercise impairment in mice. Acta Physiol..

[31] Kimmel A.R., Sztalryd C. (2014). Perilipin 5, a lipid droplet protein adapted to mitochondrial energy utilization. Curr. Opin. Lipidol..

[32] Glatz J.F.C., Luiken J. (2018). Dynamic role of the transmembrane glycoprotein CD36 (SR-B2) in cellular fatty acid uptake and utilization. J. Lipid Res..

[33] Nickerson J.G., Alkhateeb H., Benton C.R., Lally J., Nickerson J., Han X.X., Wilson M.H., Jain S.S., Snook L.A., Glatz J.F.C. (2009). Greater transport efficiencies of the membrane fatty acid transporters FAT/CD36 and FATP4 compared with FABPpm and FATP1 and differential effects on fatty acid esterification and oxidation in rat skeletal muscle. J. Biol. Chem..

[34] Furuhashi M., Saitoh S., Shimamoto K., Miura T. (2014). Fatty Acid-Binding Protein 4 (FABP4): Pathophysiological Insights and Potent Clinical Biomarker of Metabolic and Cardiovascular Diseases. Clin. Med. Insights Cardiol..

[35] Bonnefont J.P., Djouadi F., Prip-Buus C., Gobin S., Munnich A., Bastin J. (2004). Carnitine palmitoyltransferases 1 and 2: Biochemical, molecular and medical aspects. Mol. Asp. Med..

[36] Choi C.S., Befroy D.E., Codella R., Kim S., Reznick R.M., Hwang Y.J., Liu Z.X., Lee H.Y., Distefano A., Samuel V.T. (2008). Paradoxical effects of increased expression of PGC-1alpha on muscle mitochondrial function and insulin-stimulated muscle glucose metabolism. Proc. Natl. Acad. Sci. USA.

[37] Unger R.H., Clark G.O., Scherer P.E., Orci L. (2010). Lipid homeostasis, lipotoxicity and the metabolic syndrome. Biochim. Biophys. Acta.

[38] Guo Y., Cordes K.R., Farese R.V., Walther T.C. (2009). Lipid droplets at a glance. J. Cell Sci..

[39] Koves T.R., Sparks L.M., Kovalik J.P., Mosedale M., Arumugam R., DeBalsi K.L., Everingham K., Thorne L., Phielix E., Meex R.C. (2013). PPARγ coactivator-1α contributes to exercise-induced regulation of intramuscular lipid droplet programming in mice and humans. J. Lipid Res..

[40] Greenberg A.S., Coleman R.A., Kraemer F.B., McManaman J.L., Obin M.S., Puri V., Yan Q.W., Miyoshi H., Mashek D.G. (2011). The role of lipid droplets in metabolic disease in rodents and humans. J. Clin. Investig..

[41] Bonifacio A., Sanvee G.M., Bouitbir J., Krähenbühl S. (2015). The AKT/mTOR signaling pathway plays a key role in statin-induced myotoxicity. Biochim. Biophys. Acta.

[42] Lin J., Wu H., Tarr P.T., Zhang C.Y., Wu Z., Boss O., Michael L.F., Puigserver P., Isotani E., Olson E.N. (2002). Transcriptional co-activator PGC-1 alpha drives the formation of slow-twitch muscle fibres. Nature.

[43] Yokoyama M., Seo T., Park T., Yagyu H., Hu Y., Son N.H., Augustus A.S., Vikramadithyan R.K., Ramakrishnan R., Pulawa L.K. (2007). Effects of lipoprotein lipase and statins on cholesterol uptake into heart and skeletal muscle. J. Lipid Res..

[44] Rao X., Huang X., Zhou Z., Lin X. (2013). An improvement of the 2^(-delta delta CT) method for quantitative real-time polymerase chain reaction data analysis. Biostat. Bioinform. Biomath..

[45] Rothman K.J. (1990). No adjustments are needed for multiple comparisons. Epidemiology.

