# Supplementary material for: Effects of Simvastatin on Lipid Metabolism in Wild-Type Mice and Mice with Muscle PGC-1α Overexpression
Source: Int J Mol Sci. 2021 May 7;22(9):4950. doi: 10.3390/ijms22094950 (PMC8125015; doi:10.3390/ijms22094950)
Supplement: Supplementary file 1 [file ijms-22-04950-s001.zip › ijms-1163101-supplementary.pdf]

International Journal of *Molecular Sciences*

MDPI

Supplement

Effects of simvastatin on lipid metabolism in wild type mice and mice with muscle PGC-1α overexpression

Miljenko V. Panajatovic [1,2], Francois Singh [1], Stephan Krähenbühl [1], and Jamal Bouitbir [3,*]

[1] Division of Clinical Pharmacology & Toxicology, University Hospital of Basel, Switzerland m.panajatovic@unibas.ch (M.V.P); fzsingh@dundee.ac.uk (F.S); stephan.kraehenbuehle@unibas.ch (S.K.)
[2] Division of Pharmaceutical Technology, Department of Pharmaceutical Sciences, University of Basel, Switzerland
[3] Division of Molecular and Systems Toxicology, Department of Pharmaceutical Sciences, University of Basel, Switzerland; jamal.bouitbir@unibas.ch (J.B.)
* Correspondence: jamal.bouitbir@unibas.ch; Tel.: +41 61 207 6290

Citation: Panajatovic, M.V.; Singh, F.; Krähenbühl, S.; Bouitbir, J. Effects of Simvastatin on Lipid Metabolism in Wild-Type Mice and Mice with Muscle PGC-1α Overexpression. *Int. J. Mol. Sci.* **2021**, *22*, 4950. https://doi.org/110.3390/ijms22094950

Academic Editor: Gaetano Villani
Received: 14 March 2021
Accepted: 1 May 2021
Published: 7 May 2021

Publisher's Note: MDPI stays neutral with regard to jurisdictional claims in published maps and institutional affiliations.

Copyright: © 2021 by the authors. Submitted for possible open access publication under the terms and conditions of the Creative Commons Attribution (CC BY) license (http://creativecommons.org/licenses/by/4.0/).

International Journal of
Molecular Sciences

MDPI

Suppl. Fig. 1

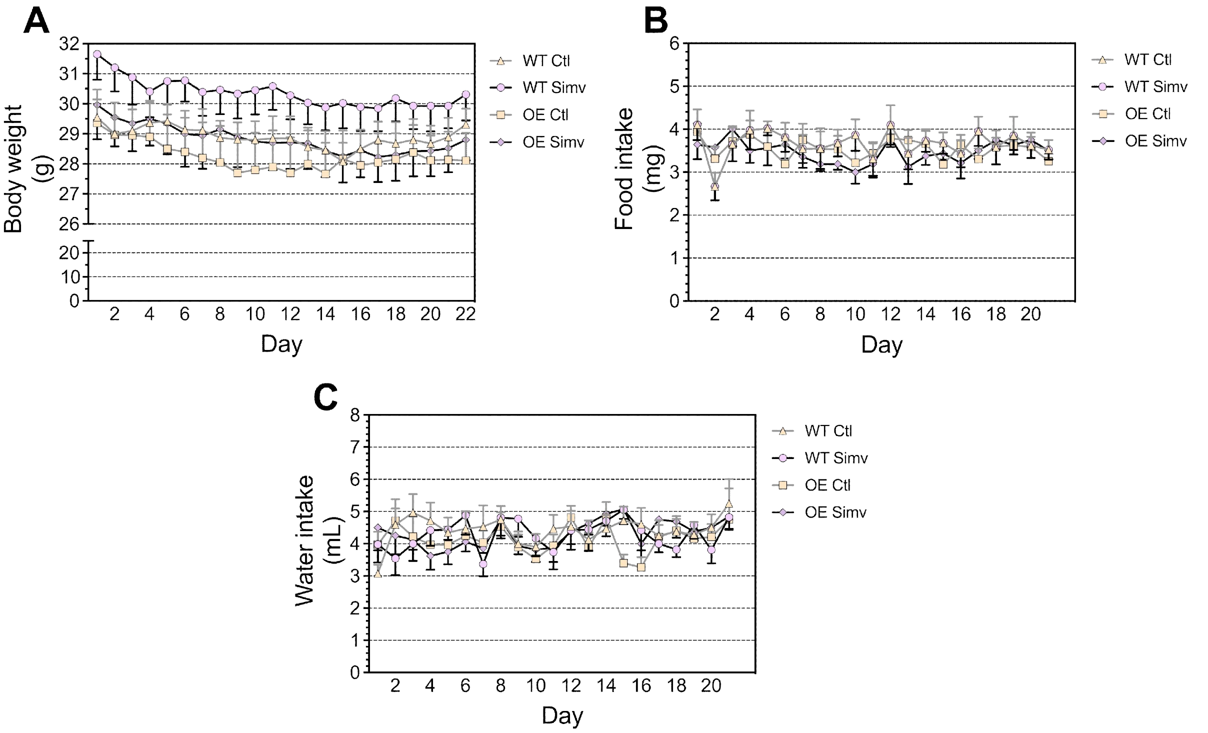

Supplementary figure 1. *Physiological characteristics over the course of the treatment*. To monitor the welfare and possible differences between groups, body weight (A), food intake (B), and water intake (C) were followed over the course of 21 days of treatment. Data are presented as mean ± SEM of 10 animals per group. Symbols on the graphs are as follows: Ctl, control; OE, PGC-1α overexpressing mice; Simv, simvastatin; WT, wild type

International Journal of
Molecular Sciences

Suppl. Fig. 2

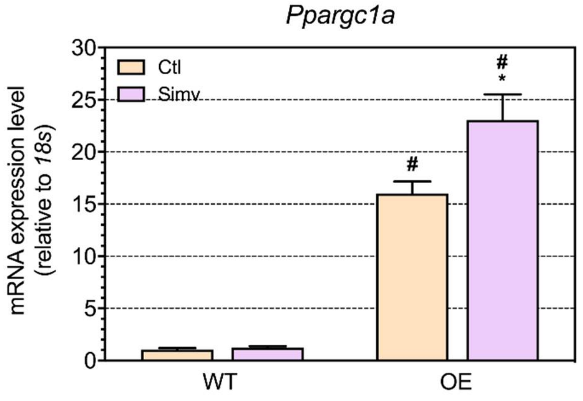

Supplementary figure 2. *Expression of PGC-1α mRNA in skeletal muscle*. Switch from glycolytic to oxidative metabolism is regulated by PGC-1α, which leads to increase in mitochondria number, and increase in fatty acid uptake and metabolism. We assessed the muscle mRNA expression of peroxisome proliferator activated receptor gamma coactivator 1 alpha (*Ppargc1a)*. Data are presented as mean ± SEM of 8 animals per group. After two-way ANOVA analysis, the treatment factor, the animal model factor, and the interaction between treatment and animal model was significant. Symbols on the graphs are as follows: * $p < .05$ simvastatin-treated vs. respective water-treated (control) mice; and # $P < .05$ PGC-1α OE vs. WT mice of the same treatment group (water or simvastatin). Ctl, control; OE, PGC-1α overexpressing mice; Simv, simvastatin; WT, wild type

International Journal of
Molecular Sciences

MDPI

Suppl. Fig. 3

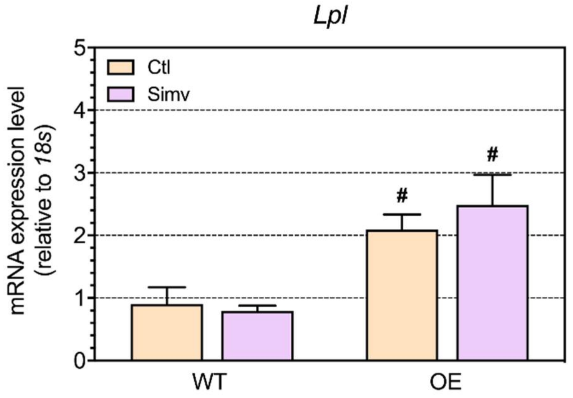

Supplementary figure 3. *mRNA expression of lipoprotein lipase (Lpl) in skeletal muscle*. Lipoprotein lipase hydrolyzes triglycerides from circulating low or very low-density lipoproteins. Data are presented as mean ± SEM of 8 animals per group. After two-way ANOVA analysis, the animal model factor was significant. Symbols on the graphs are as follows: *P < .05 between simvastatin-treated and respective control mice; and # P < .05 PGC-1α OE vs. WT mice of the same treatment group (water or simvastatin). Ctl, control; OE, PGC-1α overexpressing mice; Simv, simvastatin; WT, wild type
